# Monitoring Silane Sol-Gel Kinetics with In-Situ Optical Turbidity Scanning and Dynamic Light Scattering

**DOI:** 10.3390/molecules24162931

**Published:** 2019-08-13

**Authors:** Abul Bashar Mohammad Giasuddin, David W. Britt

**Affiliations:** Department of Biological Engineering, Utah State University, Logan, UT 84322-4105, USA

**Keywords:** organosilane, fluorosilane, ORMOSIL, hydrophobic nanoparticle, aqueous sol-gel synthesis, Turbiscan

## Abstract

Organosilanes (e.g., R’-SiOR_3_) provide hydrophobic functionality in thin-film coatings, porous gels, and particles. Compared with tetraalkoxysilanes (SiOR_4_), organosilanes exhibit distinct reaction kinetics and assembly mechanisms arising from steric and electronic properties of the R’ group on the silicon atom. Here, the hydrolysis and condensation pathways of n-propyltrimethoxy silane (nPM) and a tri-fluorinated analog of nPM, 3,3,3-trifluoropropyl trimethoxy silane (3F), were investigated under aqueous conditions at pH 1.7, 2.0, 3.0, and 4.0. Prior to hydrolysis, 3F and nPM are insoluble in water and form a lens at the bottom (3F) or top (nPM) of the solutions. This phase separation was employed to follow reaction kinetics using a Turbiscan instrument to monitor hydrolysis through solubilization of the neat silane lens while simultaneously tracking condensation-induced turbidity throughout the bulk solution. Dynamic light scattering confirmed the silane condensation and particle aggregation processes reported by the turbidity scanning. Employing macroscopic phase separation of the starting reactants from the solvent further allows for control over the reaction kinetics, as the interfacial area can be readily controlled by reaction vessel geometry, namely by controlling the surface area to volume. In-situ turbidity scanning and dynamic light scattering revealed distinct reaction kinetics for nPM and 3F, attributable to the electron withdrawing and donating nature of the fluoro- and organo-side chains of 3F and nPM, respectively.

## 1. Introduction 

Tri-alkoxysilanes, R’Si(OR)_3_, are widely employed to introduce functionality via the R’ chemistry into sol-gel processes, with specific applications as adhesion promoters and cross-linkers, as resin precursors, and to capture and encapsulate biological molecules [1,2]. For alkyl or aryl R’-groups, the resulting particles or gel are referred to as organically modified silicas (ORMOSILs). When fluorine is present on the R’ chain, they may be referred to as F-ORMOSILs. The hydrophobic alkyl- and fluoro R’ groups lower the surface energy of the resulting sol-gel based thin films, gels, or particles, and have been widely explored to create non-reflective, non-fouling, and superhydrophobic materials [3,4,5,6,7,8,9,10,11]. 

The hydrolysis and condensation chemical reactions generally occur through nucleophilic substitution reactions, with water attacking the Si center to liberate alcohol. Methoxy and ethoxy moieties are the two most common alkoxy leaving groups, and their hydrophobicity renders alkyl- and fluoro-silane precursors insoluble in aqueous solution. An R’ alkyl- or fluoro-group on the silane further reduces solubility, and an organic cosolvent, such as ethyl alcohol, is frequently employed to improve solubility and control reaction pathways. The sol-gel reactions for a trialkoxy ORMOSIL are depicted as: 

R’Si(OR)_3_ + nH_2_O

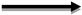

R’Si(OR)_3−n_(OH)_n_ + nROHHydrolysis(1)R’Si-OR + HO-SiR’

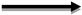

R’Si-O-SiR’ + ROHAlcohol condensation(2)R’Si-OH + HO-SiR’

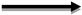

R’Si-O-SiR’ + H_2_OWater condensation(3)

**Figure 1:**
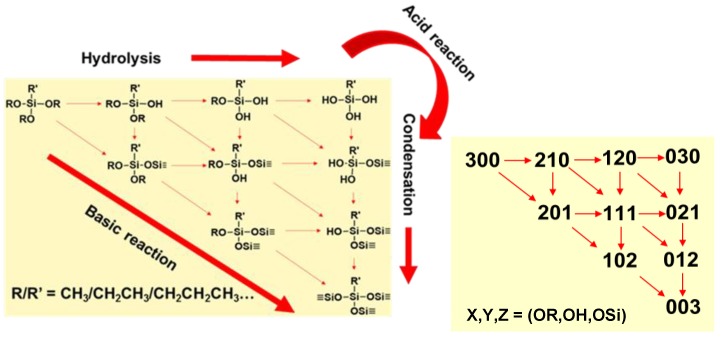
Schematic of the reaction scheme of hydrolysis and condensation kinetics of tri-alkoxysilanes under acidic and basic conditions in aqueous media, along with a numerical reaction scheme [11]. Here, (300), (030), and (003) represent the -OR, -OH, and -OSi functionalities, respectively, around a single Si atom. The neat silane consists of three alkoxy groups (300), which under acidic conditions are sequentially hydrolyzed to three hydroxyl groups (030), which may subsequently condense to produce three siloxane bonds around the Si center (003).

At a minimum, for complete hydrolysis, three moles of water are required per mole of trialkoxysilane. Excess water, along with acid catalysis, favors full hydrolysis (030) prior to initiation of condensation. Controlling the reaction rates of tri-alkoxysilane hydrolysis and condensation affords control over the size and morphology of polycondensation products that assemble into nanoparticles (sol) that may undergo growth/aggregation into larger particles and form gels depending on reaction conditions, such as acid or base catalysis, as illustrated in the reaction diagram in Figure 1. In the reaction scheme, (x,y,z) report the status of hydrolysis and condensation 

On a given Si atom, x indicates the number of alkoxy (-OR) leaving groups present, y the number of (-OH) groups, and z the number of (-O-Si) groups, as depicted in the schematics. Thus, for a trialkoxysilane under acid catalysis, the precursor (300) follows a stepwise hydrolysis to form (210) to (120), and (030) products. Under basic conditions, condensation pathways are promoted, yielding higher dimensional products earlier in the reaction. The reaction schematics shown for trialkoxysilane precursors that yield ORMOSIL and F-ORMOSIL are also descriptive of tetraalkoxysilanes, e.g., (400). While the same rules for acid and base catalysis apply, there are some key distinctions. 

The sol-gel reactions and final products formed from alkyl- and fluoro-trialkoxysilanes are distinct from tetraalkoxysilanes. First, and most notably, the chemical cross-linking capacity to form siloxane (Si-O-Si) bonds is reduced. Thus, for complete condensation (003), the chemical structure is R’SiO_1.5_, referred to as a silsesquioxane, in contrast to the SiO_2_ chemistry for a fully condensed tetraorthosilicate (004). Second, hydrophobic interactions (Si-R’ ~ ‘R-Si) influence the silane solubility and physical stabilization that is highly dependent on solvent, which in turn influences sol-gel kinetics. For example, in aqueous solution, the fully hydrolyzed (030) product exhibits an amphiphilic character, which, depending on R’ chain length, may promote self-assembly that is not possible for tetraalkoxysilanes [12,13]. Third, the steric and electronic properties of the R’ group on the silicon atom influence nucleophilic substitution. Both hydrolysis and condensation reaction rates will be reduced if the R’ group restricts access to the Si atom, thus hindering nucleophilic attack by water (reaction 1) and silanol (reactions 2 and 3). However, through inductive effects, the R’ group can stabilize partial and formal charges that may exist in transition states (TS) during these same nucleophilic substitution reactions. When the R’ group contains electron-withdrawing fluorines, a negatively charged TS would be stabilized as the charge is distributed away from the reaction center. In contrast, an electron providing an R’ group accelerates silane hydrolysis under acidic conditions, where a positively charged TS may arise, but slows hydrolysis under base catalysis due to the anticipated negatively charged TS [14]. Thus, while both fluoro- and alkyl-R’ groups are employed to introduce a hydrophobic character into the resulting condensation product, the electron donating ability of an alkyl R’ group vs. the electron withdrawing nature of a fluoro-containing R’ group lead to differences in reaction rates, which in turn may affect the sol-gel kinetics and thus the size and polydispersity of the condensation products, as well as the porosity and surface area of gels.

**Figure 2:**
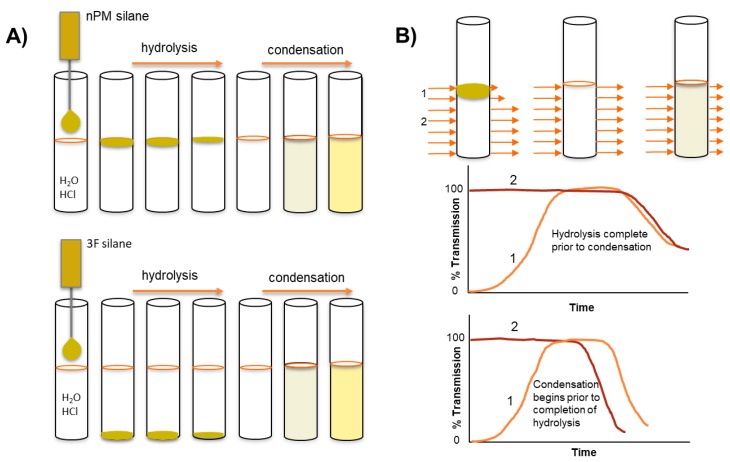
(**A**) Schematic of n-propyltrimethoxy silane (nPM) and 3,3,3-trifluoropropyl trimethoxy silane (3F) silane sol-gel reaction in aqueous solution catalyzed by weak acid. (**B**) Turbiscan height profile of reaction tube monitors the depletion of neat silane through hydrolysis and subsequent particle formation (sol) through condensation-induced turbidity. Reaction conditions can be tuned to favor completion of hydrolysis prior to the onset of condensation. Continuous scanning of the reaction tube provides transmission changes at defined height and time intervals.

In the present work, ORMOSIL and F-ORMOSIL sol-gel kinetics are investigated at acidic pH in water without a cosolvent, thus creating an interface between the neat silane and the aqueous reaction solution. The macro silane/water interface is used here to track the hydrolysis and condensation of the ORMOSIL, n-propyltrimethoxy silane (nPM), and the F-ORMSIL analog, 3,3,3-trifluoropropyl trimethoxy silane (3F), as illustrated in Figure 2. During hydrolysis, the solubility balance is tipped as the hydrophobic alkoxy leaving groups are replaced with silanol moieties, thus depleting the neat silane that resides as an insoluble phase on top of the water for nPM, and at the bottom of the reaction vessel for the denser 3F. This two-phase reaction system permits in-situ light transmission analysis of hydrolysis and condensation, using turbidity scanning to monitor transmitted and reflected light at defined height increments in the reaction tube. Hydrolysis is reported as an increase in transmission through depletion of the oily lens of neat silane precursor. Condensation correlates to a decrease in transmission as a cloudy sol is formed. Condensation is further confirmed in-situ using dynamic light scattering. Distinct reaction kinetics and assembly mechanisms are observed for nPM and 3F that are attributed to differences in steric and electronic properties of the respective alkyl- or fluoro-groups attached to the silicon atom. At the investigated pH values of 1.7, 2, 3, and 4, these techniques reveal a faster hydrolysis for nPM than 3F, with an opposite trend observed during condensation at the same pH. 

## 2. Results and Discussion

**Figure 3:**
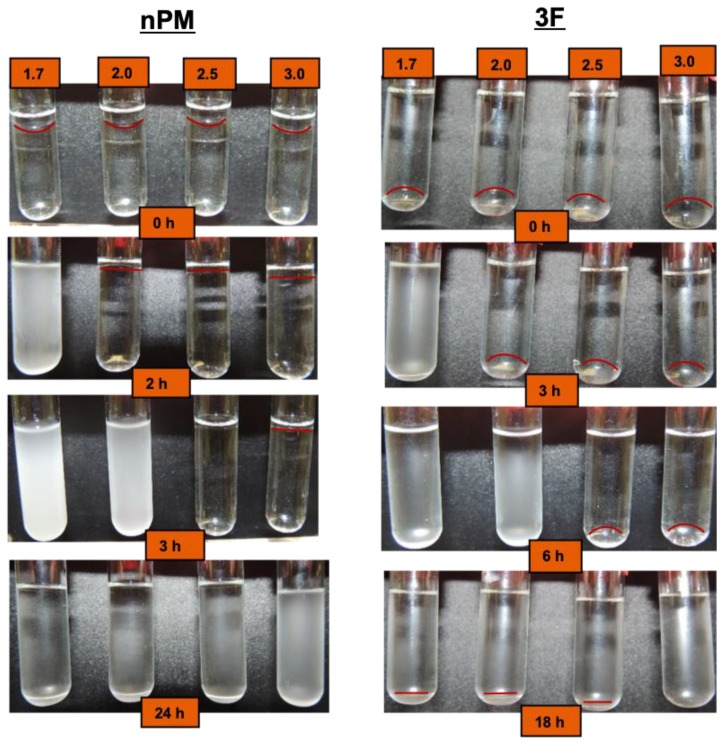
Time-lapse observation of nPM (0.4 M) (**left** images) and 3F (0.4 M) (**right** images) hydrolysis and condensation at pH 1.7, 2.0, 2.5, and 3.0. Equal volumes of the two silanes were used, with nPM floating on top of the aqueous solution and 3F dropping to the bottom. Hydrolysis depletes the neat silane lenses, followed by onset of condensation and turbidity due to light scattering by the sol. Aggregation of the particles in the sol clears the solution as particles drop to the bottom of the reaction tubes.

The depletion of the immiscible silane precursor and evolution of a turbid sol for nPM and 3F can be visually assessed through snapshots of reaction vials at defined timepoints as shown in Figure 3. The neat silanes are immiscible in water and form visible lenses at the top (nPM) and bottom (3F) of the reaction tubes. The depletion of the lenses as hydrolysis occurs is difficult to discern with the eye, and the boundaries between neat silane and bulk solution are highlighted in the photos. The turbidity associated with condensation is clearly seen, and the expected trend of an increased rate of hydrolysis and condensation is observed with decreasing pH. 

Acid-catalyzed hydrolysis occurs through a protonation step of the alkoxide group, yielding Si-(ORH)^+^, promoting nucleophilic attack by water on silicon and forming a pentavalent transition state with the S_N_2 character. In ORMOSILs, R’Si(OR)_3_, the electron donating R’ group, stabilizes the positive charge character of the transition state, and thus accelerates hydrolysis relative to tetraorthosilicates (Si(OR)_4_) [14]. With each hydrolysis step, the transition state is destabilized through the greater electron withdrawing nature of -OH compared to -OR. Similarly, the electron withdrawing nature of the trifluoro moiety in the 3F silane destabilizes the transition state, thus acid-catalyzed 3F hydrolysis is expected to be slower than nPM hydrolysis, as discernible in Figure 3. This is most evident for the nPM pH 2.5 vials at the 3 h reaction time, where the lens of neat silane has disappeared completely, whereas at the same pH, the 3F lens is still present at 3 h.

**Figure 4:**
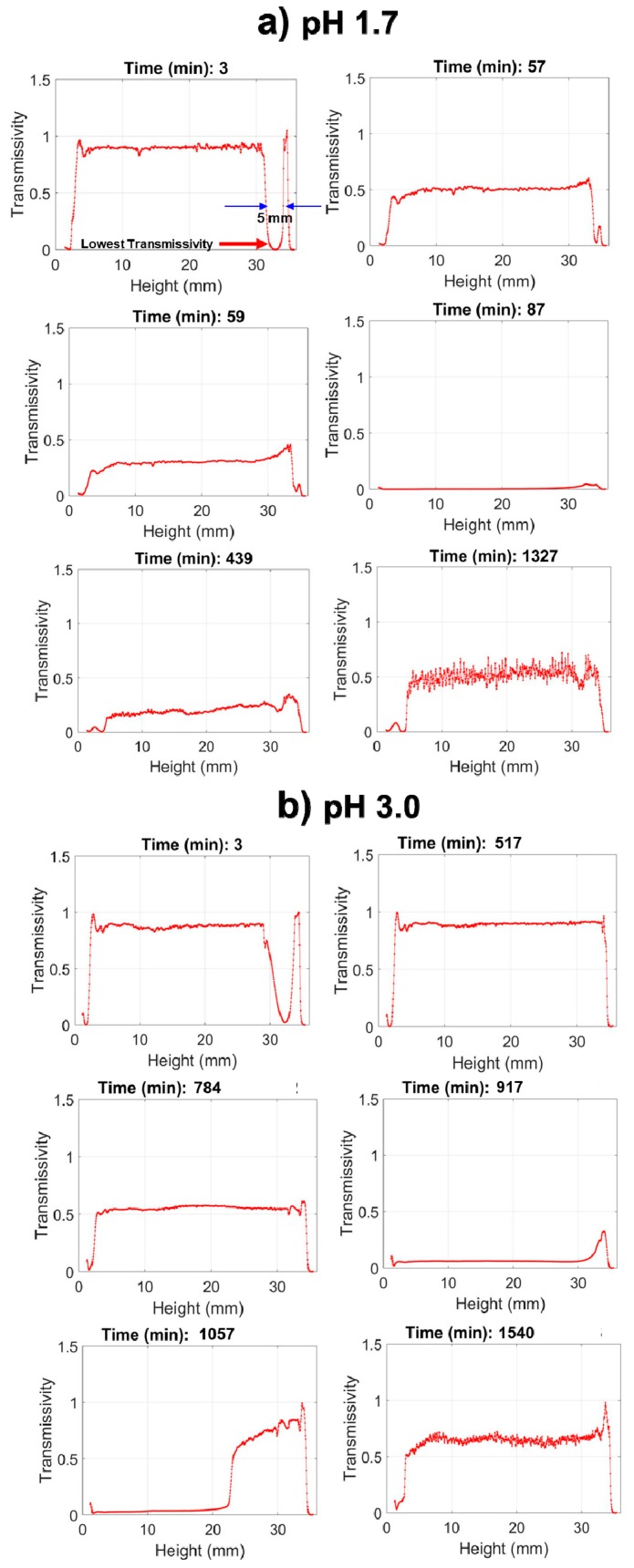
Turbidity scanning of the sol-gel reaction for nPM in water at pH 1.7 (**a**) and pH 3.0 (**b**). The graphs show light transmission vs. tube height, with 0 mm representing the bottom of the reaction tube. The lens of neat silane occupies the top ~5 mm of the 35 mm tube height, causing the dip in transmission seen at early times near the top of the tube. Hydrolysis solubilizes the silane, and the thickness of the lens narrows with time. Condensation in the bulk is observed by the drop in light transmission through the entire length of the tube.

To precisely measure hydrolysis and condensation, the optical density, or turbidity, of the solution can be measured at defined heights. A conventional spectrophotometer measures at a fixed height; however, the Turbiscan instrument measures light transmission (and reflection) at defined increments throughout the full height of the tubes, as illustrated schematically in Figure 2B [15] This allows for a near simultaneous assessment of hydrolysis (lens depletion) and condensation (turbidity evolution) with a resolution of several seconds per scan. In Figure 4, select transmission profiles produced for nPM at pH 1.7 and 3.0 are provided to illustrate the procedure. The neat silanes are transparent and transmit incident light; however, where the silane meets the water, the incident light is scattered due to formation of a curved lens at this interface, thus transmission is greatly reduced at this solubility boundary. In bulk solution, the hydrolyzed silanes have no influence on light transmission, and only upon polycondensation and particle formation does light scattering reduce transmission through the bulk. Thus, Turbiscan measures hydrolysis through the retreat of the reaction interface as insoluble neat silane is converted to soluble hydrolyzed silane while also measuring polycondensation and phase separation through the evolution of scattering in the bulk. This is a unique application of the instrument for sol-gel reaction chemistry, allowing for relative reaction rate comparisons.

The Turbiscan data in Figure 4 reveal that at pH 1.7, nPM condensation begins by 66 min, indicated by a uniform drop in transmissivity throughout the tube, yet hydrolysis is not complete as indicated by a near zero transmissivity at the top (30–35 mm) of the tube due to scattering at the neat silane/solution interface. The condensation rate is rapid, forming a completely opaque suspension within 20 min after onset as indicated by the zero light transmission at 86 min. In contrast, at pH 3.0, nPM hydrolysis required over 500 min to reach completion in which the lens dissolved, and transmission was even throughout the entire tube. At this less acidic pH, the hydrolyzed silane is also very stable, with onset of condensation delayed until after 700 min, and maximum turbidity of the sol obtained after 916 min. Particle aggregation and settling processes are also readily discerned from the scans as an increase in transmission at later times. This is evident for nPM at pH 3.0, where a “wave” of clearing starts at the top of the tube at 916 min and moves down the tube.

Animations of the collected data over all times are provided in the Supporting Information files, allowing the hydrolysis, condensation, and aggregation/settling processes to be clearly visualized and contrasted for pH 1.7, 2.0, 3.0, and 4.0. (Videos S1, S2, S3, and S4 in Supplementary Materials) Evidence of a secondary particle growth process is seen in the pH 2.0 transmission data animation, where following an initial condensation and particle settling process, turbidity increases uniformly in the bulk, followed by settling. These processes are only observed by scanning the entire reaction tube height, an advantage afforded by using the Turbiscan instrument in contrast to a standard spectrophotometer.

**Figure 5:**
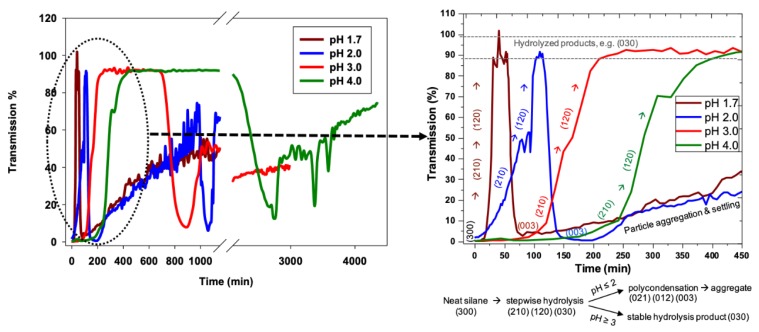
Hydrolysis and condensation kinetics of nPM (0.4 M) at pH 1.7, 2.0, 3.0, and 4.0. Transmissivity data were taken at the height where transmissivity is the lowest at the beginning due to the presence of nPM. The right panel shows the transmission during the first 450 min, with putative (xyz) hydrolysis and condensation process nomenclature according to Figure 1.

By selecting the transmission at a fixed height near the top of the nPM lens (e.g., at a tube height of 34 mm) to be monitored, the kinetics of the sol-gel processes can be compared, as shown in Figure 5. However, the onset of condensation prior to complete hydrolysis (e.g., pH 1.7) cannot be detected by monitoring at a fixed height. Once the lens is dissolved through full hydrolysis, condensation-induced turbidity occurs uniformly throughout the tube. The kinetics for nPM hydrolysis and condensation for nPM at the four pH values provide information through the time of onset of the respective hydrolysis and condensation events, as well as the slopes for each process. Figure 5 shows the Turbiscan transmission data that reflect the hydrolysis and condensation kinetic profiles of nPM at pH 1.7, 2.0, 3.0, and 4.0. The transmissivity was measured at a fixed height of 34 mm in the tube, where the nPM lens resulted in a minimum transmission value. At pH 1.7, rapid hydrolysis of the lens eliminated scattering and increased transmissivity to 100% within 45 min, followed by an equally rapid condensation-induced transmissivity drop to 2% after 80 min. This is expected based on the 2 h snapshot of the pH 1.7 reaction tube in Figure 3. By 900 min, the transmissivity increased to 50% as condensed particles aggregated and settled from the solution, which can also be seen in the 24 h snapshot of nPM at pH 1.7 in Figure 3.

Increasing the pH above 1.7 (e.g., 2.0, 3.0, or 4.0) delays the onset of hydrolysis, decreases the hydrolysis rate (lower initial slopes), and extends the stability of the hydrolyzed product (high transmissivity plateau) as seen in Figure 5. These data demonstrate turbidity scanning as a facile means to evaluate the key sol-gel steps of: 1) Neat silane hydrolysis, 2) hydrolysis product stability, followed by 3) condensation and 4) subsequent particle aggregation, settling, and resuspension events. The transmissivity patterns along the tube length during the hydrolysis and condensation of nPM are better visualized in the zoomed-in analysis of the first 450 min of the transmissivity curves in Figure 5. Under acid catalysis, ORMOSILs, such as nPM, undergo a stepwise hydrolysis process from (300) to (210), (120), and finally (030) [16,17,18]. With each step, silane solubility increases, leading to near 100% transmissivity, as seen for the nPM data. The expected influence of pH on nPM hydrolysis is seen in these data, with hydrolysis complete by 50 min at pH 1.7, 100 min at pH 2.0, 200 min at pH 3, and 350 min at pH 4.0. Likewise, hydrolyzed nPM stability is highly sensitive to reaction pH, increasing from ~10 min stability at pH 2.0 to over 24 h at pH 4.0. Aggregation of the condensed species leads to particle settling and a further increase in transmissivity, which is observed at each of the investigated pH values.

**Figure 6:**
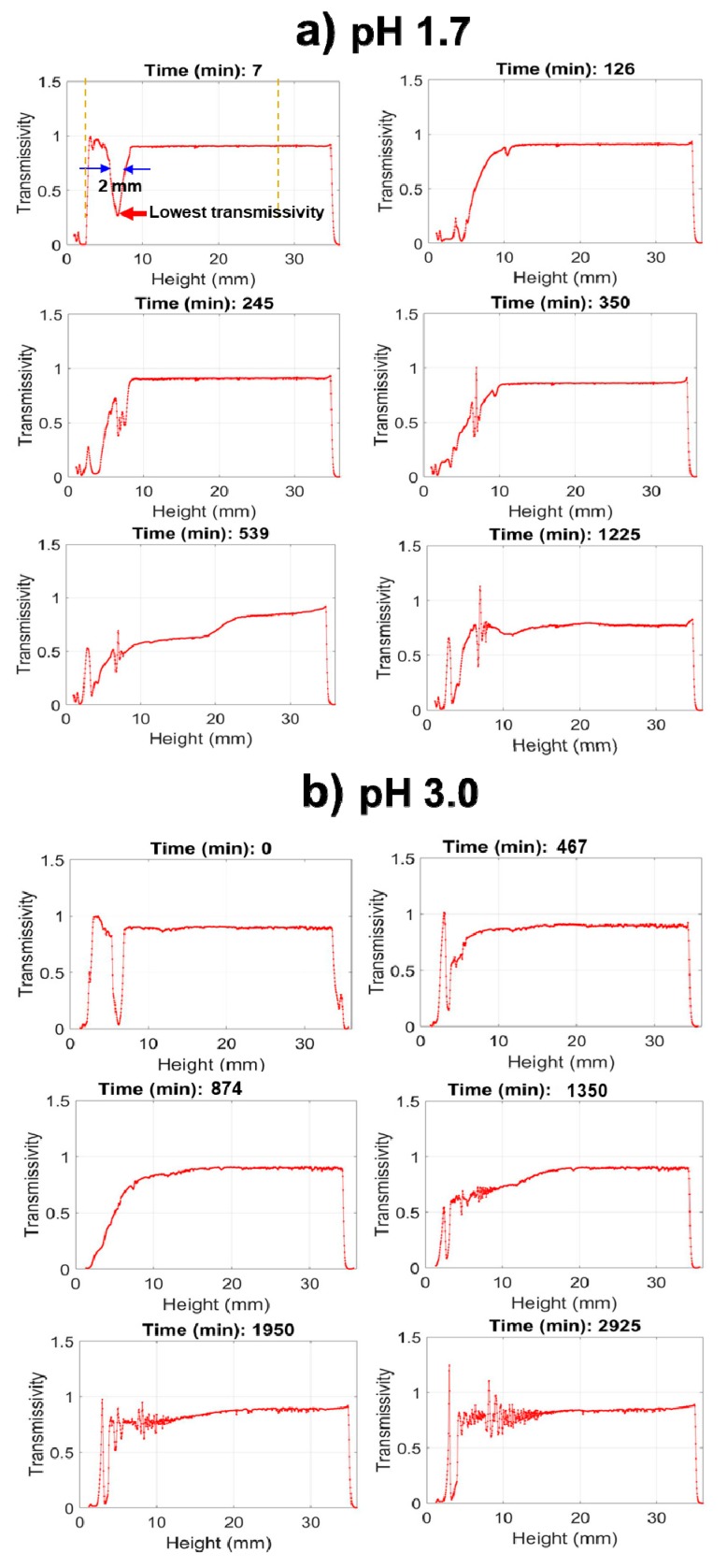
Turbidity scanning of the sol-gel reaction for 3F in water at pH 1.7 (**a**) and pH 3.0 (**b**). The graphs show light transmission vs. tube height, with 0 mm representing the bottom of the reaction tube. The lens of neat silane occupies the bottom ~2 mm of the 35 mm tube height, causing the dip in transmission seen at early times near the bottom of the tube. Hydrolysis solubilizes the silane, and the lens thickness narrows with time. Condensation in the bulk is observed by the drop in light transmission initially at the bottom and later through the entire length of the tube.

Silanes that are denser than water, such as 3F, can also be measured using Turbiscan, as shown in Figure 6 for the full tube height transmissivity scans, which report hydrolysis and condensation profiles of 3F at pH 1.7 and pH 3.0. As seen in Figure 6, 100% transmissivity is observed throughout the solution except at the tube bottom, where neat 3F created a 2 mm thick lens that scattered the incident light, thus reducing transmitted light. As with nPM, 3F also shows an increase in hydrolysis and condensation reaction rates with decreasing pH that are evident in these snapshots of the transmission scans. The full set of scans can be viewed as an animation in Video S5, S6, S7, and S8, more clearly revealing secondary processes of condensation product aggregation and settling as reported by drops in turbidity throughout the tube. Following decreases in turbidity indicating particle settling, 3F, like nPM, shows subsequent turbidity increases, which may reflect secondary nucleation and growth events, or resuspension of aggregates that separate due to charge repulsion. 

**Figure 7:**
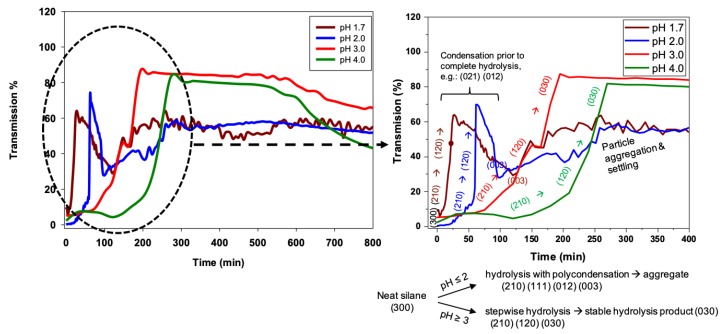
Hydrolysis and condensation kinetics of 3F (0.4 M) based on transmissivity, using Turbiscan-analyzed data at pH 1.7, 2.0, 3.0, and 4.0. Transmissivity data for different pH are taken at the height where transmissivity is the lowest at the beginning due to the presence of 3F. A zoomed figure in the right shows how the chemical transformation occurred in 3F during the hydrolysis and condensation process.

By monitoring the transmission at a fixed height near the bottom of the 3F lens (e.g., 6 mm from bottom), where lowest transmissivity appears, the kinetics of the sol-gel processes can be compared at the different pH values as shown in Figure 7. The lens curvature leads to light scattering and reduced transmissivity; where the lens meets the tube wall, it forms a cylinder and the transmissivity therefore increases again below this curved interface of the lens with the water. At pH 1.7, the transmissivity measured at this fixed height increased from 5% at the start of the reaction to about 65% at 30 min, followed by a decrease in transmission. In contrast to nPM, maximum transmissivity was never more than about 65% for 3F at pH 1.7, indicating that under these conditions, 3F was likely not fully hydrolyzed prior to the onset of condensation; however, at higher pH (e.g., 3.0 or 4.0), stable hydrolyzed 3F species were attained. The hydrolyzed 3F lifetime is greatest at pH 3.0, unlike the hydrolyzed nPM, which is highest at pH 4.0. Also, the shortest hydrolyzed 3F lifetime is at pH 2, whereas it is at pH 1.7 for hydrolyzed nPM. Based on the findings from the Turbiscan analysis as discussed, a brief summary of the optimum hydrolysis, condensation, sedimental time, and the stability period of the hydrolysis state is presented in Table 1. 

3F is more acidic than nPM and hydrolyzed faster. For both silanes, the data show that the lifetime of the anticipated hydrolyzed species (high transmissivity plateau) is increased as pH is increased. Under mildly acidic conditions, hydrolyzed silanes are stable in a manner highly dependent on the side group. For bulky R’ groups, such as a phenyl ring, the silanol stability is enhanced due to steric hindrance against nucleophilic substitution [19]. For base-catalyzed sol-gel reactions, both hydrolysis and condensation occur in parallel, thus condensation occurs via the alcohol-liberating condensation pathway, as depicted in reaction (2). The selection of acid or base catalysts influences the resulting condensation and gel formation, with more porous gels resulting for base-catalyzed pathways and denser gels for acid catalyzed pathways [20].

**Figure 8:**
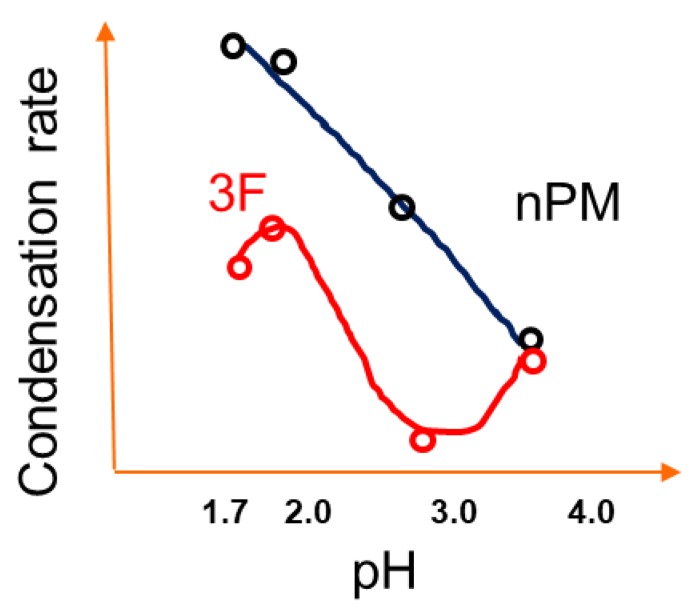
pH based condensation trends of nPM and 3F identified from Turbiscan.

The pH trends for 3F condensation rates are also distinct as compared to nPM. The 3F condensation rate is faster at pH 2 than pH 1.7, and the condensation rate at pH 4.0 is faster than that of pH 3.0. These distinctions in the nPM and 3F condensation trends at the investigated pH values are schematically depicted in Figure 8. The distinctions in the sol-gel kinetics are attributable to the electron withdrawing and donating nature of the fluoro- and organo-side chains of 3F and nPM, respectively. Inductive effects can significantly shift the pKa of acids. Distinct isoelectric points of ORMOSIL and F-ORMOSIL analogs, such as nPM and 3F, are expected. As reviewed by Coltrain and Kelts, silane condensation is specifically acid and base catalyzed: Above the silica isoelectric point of ~pH 2.5, condensation proceeds by nucleophilic attack of deprotonated silanols on neutral silicates, while below the isoelectric point, the reaction proceeds by protonation of silanols followed by electrophilic attack [21]. 

Due to electron-donating inductive effects, the isoelectric point for nPM was expected to be at least 4.5 [22], and our data for nPM agree with acid-specific catalysis of condensation as the pH decreased from 4.0 to 1.7. For 3F, the presence of the highly electronegative fluorines lowers the isoelectric pH relative to the non-fluorinated nPM. TEOS has a point of zero charge near 2.2, and 3F appears to be between this and that of nPM, as the fluorines are three carbons removed from the silicon center, so the electron withdrawing inductive effect is diminished. These trends are depicted in Figure 8.

**Figure 9:**
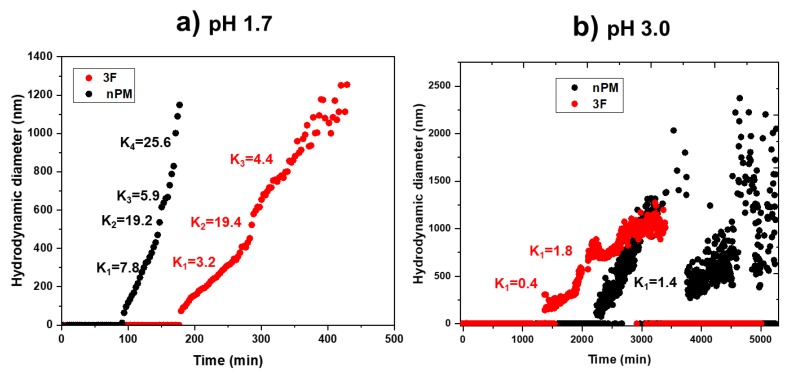
Comparison of the hydrodynamic sizes of the colloidal particles evolved during the hydrolysis and condensation of nPM (0.1 M) and 3F (0.1 M) at pH 1.7 (**a**) and pH 3.0 (**b**). Slopes are indicated for a relative comparison of particle formation rates.

To better assess the hydrolysis and condensation kinetics, dynamic light scattering (DLS) was used to monitor colloid evolution during the condensation process at pH 1.7 and 3.0. Figure 9a shows a comparative hydrodynamic size of the colloidal particles formed during the hydrolysis and condensation of 3F and nPM at pH 1.7. For nPM, light scattering corresponding to sub-100 nm particle formation was observed within 80 to 90 min. After ~180 min, the nPM particle sizes exceeded 1000 nm and the scattering signal was lost as particles aggregated and settled out of solution. Figure 9a shows a similar particle evolution pattern for 3F. The slower hydrolysis and condensation kinetics of 3F compared to nPM at pH 1.7 may reflect the distinct inductive effects shifting the isoelectric points of the silanes in opposite directions [22]. At pH 3.0, evolution of the colloidal particles was delayed compared to that at pH 1.7, as shown in Figure 9b; however, at the higher pH, particles formed in the 3F solution before the nPM solution. Also, decreases in particle sizes followed by subsequent increases are observed in Figure 9b, which may be due to the sedimentation of larger particles from solution followed by particle remodeling/regrowth phenomena.

Both the Turbiscan and DLS data reflect a crossing over of the isoelectric pH of the 3F when the pH increases from 1.7 to 3.0, arising from the electron withdrawing trifluoro-propyl side chain. For the non-fluorinated silane analog, nPM, all investigated pH values are below the isoelectric pH, which increased above 4.0 due to the electron donating character of the n-propyl group. Both nPM and 3F follow the acid-catalyzed pathway depicted in Figure 1; however, as visually observed in Figure 3, the nPM forms a highly turbid sol more rapidly than 3F due to the electron-donating, positive inductive effect of the n-propyl chain that stabilizes the positive charge on the silicon atom arising during protonation of the alkoxy leaving group. Turbiscan measures silane hydrolysis and condensation through light scattering and absorbance, respectively. Changes in the height of the light scattering event at the curved interface formed by the neat silane with water reflects the solubilization of the silanes upon hydrolysis. As shown for nPM and 3F, this can be employed with silanes that are less dense (nPM) and denser (3F) than water. Condensation leading to reduction in light transmission is simultaneously measured throughout the reaction tube, which is distinct from measuring sol formation in a standard spectrophotometer that measures at a fixed tube height. DLS can also be employed as an in-situ technique for reporting condensation but not hydrolysis.

## 3. Materials and Methods 

### 3.1. Source of Chemicals

Double distilled deionized (DI) water was collected from Barnstead^TM^ MegaPure^TM^ Glass Still (Thermo Scientific); hydrochloric acid (HCl) 1.0 N was purchased from Ricca Chemical Company (Arlington, TX, USA); 3,3,3-trifluoropropyl trimethoxy silane (3F, >95% purity, MW = 218.3, d = 1.14 g/mL) and n-propyltrimethoxy silane (nPM, >95% purity, MW = 164.3, d = 0.94 g/mL) were purchased from Gelest, Inc. (Morrisville, PA, USA); and potassium chloride (KCl) was purchased from Thermo Fisher Scientific, Fair Lawn, NJ, USA.

### 3.2. Monitoring Reaction Time through the Silane Phase

Four different 0.4 M solutions were prepared for both nPM and 3F at pH 1.7, 2.0, 2.5, and 3.0 using 20, 10, 5, and 1 mM HCl solution respectively. To prepare 0.4 M nPM and 3F solution, 2.32 mL acidic DI water was pipetted into a glass test tube (0.5 cm^2^ inner surface area) and then 0.18 mL nPM or 3F was poured in it. The hydrolysis and condensation were monitored using a Nikon COOLPIX P510 camera. 

### 3.3. Turbiscan Analysis

Hydrolysis and condensation behavior of nPM (0.4 M) and 3F (0.4 M) at pH 1.7, 2.0, 3.0, and 4.0 were followed by measuring the transmissivity of light (850 nm) through the samples using a Turbiscan instrument (Turbiscan Classic, MA200, L’Union, France), and data were analyzed using Turbisoft 1.2.1. All solutions were prepared as described in the previous paragraph, and the pH 4.0 solution was 0.1 mM HCl. All solutions were placed in the Turbiscan tubes to a height of 35 ± 0.5 mm, and transmissivity was measured as a function of time. Transmissivity was measured throughout the height of the solution in the tubes for 12 to 96 h, and measurements were taken at 2 to 22 min intervals, depending on the pH of the solutions, which influenced reaction rates. Data were plotted using Sigma Plot 10.0. Videos of the Turbiscan-obtained data were prepared using MATLAB R2018b.

### 3.4. Dynamic Light Scattering (DLS) Analysis

Evolution of polymeric NPs during nPM and 3F hydrolysis and condensation were analyzed by DLS (DynaPro NanoStar, Wyatt Technology Corporation, Santa Barbara, CA, USA), with a 658 nm laser. Measurements were taken using a quartz cuvette (JC-426, 1 µL) with 0.7 mL of nPM (0.1 M) and 3F (0.1 M) solution at pH 1.7 and 3.0. The silane concentrations of nPM (0.4 M) and 3F (0.4 M) used in the Turbiscan analysis were too high for the DLS instrument to measure single scattering events. Furthermore, the sol-gel reactions reported through DLS cannot be directly compared with those obtained from turbidity scanning due to differences in the geometries of the sample tubes and cuvettes used for Turbiscan and DLS, respectively. The data were recorded as an average of 10 five-second acquisitions. Hydrodynamic radii (R_h_) of polymeric NPs were obtained using Dynamic software (version 7.0.3, Wyatt Technology Corporation, Santa Barbara, CA, USA), where the Stokes–Einstein equation was applied to convert the intensity auto-correlation into particle sizes. Inner geometry of the quartz cuvette was circular in shape and the DLS laser light was passed through approximately 1/3 of the height of the cuvette bottom.

## 4. Conclusions

The sol-gel kinetics of a short chain alkyl-silane, nPM, and fluorinated analog, 3F, were investigated in aqueous solutions using turbidity scanning. The immiscibility of the silanes in water creates a reaction interface that scatters incident light, allowing the dissolution of the insoluble silane lens through hydrolysis to be tracked. Simultaneously, the technique tracks condensation by scanning through the height of the tube to monitor condensation product-induced light scattering in the bulk solution. The trifluoro-propyl moiety renders 3F denser than water, creating the reaction interface in the bottom of the reaction tube whereas the non-fluorinated nPM resides on top of the water. For 3F, this creates some potential interference as condensation products aggregate and settle toward the bottom of the tube. The electron-withdrawing fluoro-groups also accelerate the condensation of 3F compared to nPM. The use of optical turbidity scanning and the dynamic light scattering technique showed the distinct pattern of the hydrolysis and condensation kinetics of nPM and 3F. 

## Figures and Tables

**Table 1:** Summary of the average hydrolysis, condensation, sedimental time, and stability of the hydrolyzed state at pH 1.7, 2.0, 3.0, and 4.0 of nPM and 3F.

	pH	Average Hydrolysis Time	Condensation Starting Time	Stability of Hydrolyzed State	Average Condensation Time	Average Sedimentation Time
		min	min	min	min	min
	1.7	45	46	1	100	1000
nPM	2.0	100	110	10	200	1000
	3.0	200	700	500	900	1100
	4.0	350	1850	1500	2800	>4000
	1.7	30	30	0	120	250
3F	2.0	80	80	0	90	275
	3.0	180	600	420	>800	NA
	4.0	275	500	225	>800	NA

## Supplementary Materials

The following are available online, Video S1: nPM Turbiscan full scan at pH 1.7, Video S2: nPM Turbiscan full scan at pH 2.0, Video S3: nPM Turbiscan full scan at pH 3.0, Video S4: nPM Turbiscan full scan at pH 4.0, Video S5: 3F Turbiscan full scan at pH 1.7, Video S6: 3F Turbiscan full scan at pH 2.0, Video S7: 3F Turbiscan full scan at pH 3.0, and Video S8: 3F Turbiscan full scan at pH 4.0
